# 3R gene expression in chronic lymphocytic leukemia reveals insight into disease evolution

**DOI:** 10.1038/bcj.2016.39

**Published:** 2016-06-03

**Authors:** S Grgurevic, L Berquet, A Quillet-Mary, G Laurent, C Récher, L Ysebaert, C Cazaux, J S Hoffmann

**Affiliations:** 1 INSERM, U1037, CRCT, Toulouse, France; 2 University Toulouse III Paul Sabatier, U1037, CRCT, Toulouse, France; 3 CNRS, ERL5294, CRCT, Toulouse, France; 4 Equipe ‘Labellisée LA LIGUE CONTRE LE CANCER 2013', Toulouse, France; 5 Laboratoire d'Excellence Toulouse Cancer Labex Toucan, CRCT, Inserm U1037, CNRS ERL5294, Toulouse, France; 6 Department of Hematology, Institut Universitaire du Cancer (IUC), Toulouse, France

DNA transactions, including replication, repair of damaged DNA and recombination (the so-called ‘3Rs') are crucial processes required for preserving genome integrity and limiting cancer risk.^1^ Our previous large-scale studies performed in solid cancers displaying major genetic instabilities, demonstrated that specific misexpression of DNA replication genes could explain cancer-associated genetic changes and predict patients' outcomes; the overexpression of the unconventional DNA polymerase POLQ being, for instance, associated with poor overall survival in breast^2^ and non-small cell lung cancer.^3^

Chronic lymphocytic leukemia (CLL) is the most common leukemia in adults. Besides chromosome aberrations affecting karyotype integrity,^4^ CLL genomes are characterized by elevated frequency of nucleotide point mutations.^5^ These different manifestations of genetic instability are not mutually exclusive and may be mechanistically confined to a common pathogenic pathway, which has never been deeply explored.

The clinical course of CLL is highly heterogeneous, ranging from indolent and asymptomatic forms that can remain ‘benign' for as long as a decade, to more aggressive disease requiring immediate chemotherapeutic treatment. Estimating the course of the disease development at the diagnostic stage has historically relied on clinical staging systems, whereas the mutational status of the immunoglobulin heavy variable gene (IGHV), fluorescence *in situ* hybridization cytogenetics and presence of common somatic mutations contributed to refining of the prognostication later on.^4^ However, the former indicators do not fully explain the heterogeneity of the disease evolution before the therapeutic treatment.

Essential DNA replication processes have, surprisingly, remained an underexplored source of biomarkers and anti-cancer targets for hematological neoplasms, especially CLL, probably because such an explorative approach was not intuitive for this malignancy. Indeed, CLL has been characterized by accumulation of malignant cells resting in quiescent, mostly G0 and early G1 phase of the cell cycle.^6^ In contrast to the non-proliferative peripheral blood compartment that is used for diagnostic purposes, a small actively proliferating fraction of CLL cells residing within the lymph nodes contributes to the daily generation of the leukemic clone. However, according to the international ethical guidelines applied, samples originating from the lymph nodes are not always readily available.

Here, we reasoned that the aberrant 3R in CLL and associated occurrence of the ‘replicative stress',^7^ a driving force of chromosomal instability^8^ could represent a major source of genomic variability during the proliferation stages of CLL cells, consequently motoring the heterogeneous evolution of the disease, and could be used as a tool to understand the dynamics of evolution and the clinical outcomes of CLL patients.

CLL patient samples at diagnosis (*n*=141) and healthy donor controls (*n*=10) were obtained following an informed consent and in accordance with the Declaration of Helsinki. Patients were diagnosed with CLL according to standard clinical and laboratory criteria. Relevant clinical information regarding the cohort is summarized in Table 1. The cohort recapitulates already established correlations between known biological and clinical parameters (*P* values <0.05) (Supplementary Figure S1).

Purification of B cells from CLL and healthy donors' samples was performed by an immunomagnetic selection. Purity and viability of B-lymphocyte population was assessed by flow cytometry. RNA extraction and its quality assessment preceded complimentary DNA synthesis.

Gene expression assay was based on a custom selection of 94 probes including, among others, 3 known diagnostic markers, 1 proliferation marker and 82 3R genes (Supplementary Table S1). Detailed sample preparation and gene expression normalization are described in Supplementary Materials and Methods.

Differential gene expression was determined by performing binomial test for CLL vs healthy donor samples and the Mann–Whitney test was used to compare different CLL subgroups (Supplementary Figure S2). Prognostic value of a candidate gene was determined by Kaplan–Meier estimates, whereas marker independence was assessed by the multivariable Cox regression analysis.

We first investigated the expression of 82 genes involved in 3R DNA transactions, that is, initiation of DNA replication (firing and licensing of 50 000 human replication origins), elongation and maintenance of stability of DNA replication forks, signaling and excision/recombination repair of DNA damage, in CLL B lymphocytes compared to healthy B lymphocyte controls. In unsupervised analysis, CLL samples showed a distinct 3R profile and clustered apart from healthy donor CD19+ cells (Figure 1a). Clinical characteristics of CLL clusters are detailed in Supplementary Figure S3. Differential expression data of individual genes deregulated in CLL vs healthy donors are represented in Figure 1b and Supplementary Table S2 (all *P* values<0.001). A limited overview of the CLL replisome is illustrated in Figure 1c. Our data showed that genes coding for proteins implicated in the firing of the replication origins, namely *CDC7*, *DBF4B* and *MCM4* were overexpressed in CLL. Interestingly CDC7 inhibition has been previously proposed as a therapeutic strategy to target CLL.^9^ The overexpression of *MCM4*, on the other hand, could indicate a proliferative potential of neoplastic CLL lymphocytes residing in an early ‘in-cycle' G1 state, as it was already shown to be the case with *MCM2* expression.^6^ Among genes involved in progression and stability of replication forks, *PCNA* and a component of its alternative loading factor *DSCC1*, as well as the DNA helicase *MCM8*, lagging-strand replicative DNA polymerase *POLD1* and *ASF1A*, a component of the replication-dependent chromatin assembly, were upregulated in CLL lymphocytes. Regarding genes implicated in DNA repair processes, *HMGA1*, known to influence repair of DNA lesions, was downregulated in CLL, whereas *XRCC4*, functioning in double-strand break repair and the *RECQL* helicase were overexpressed. Among specialized translesional/repair DNA polymerases, *POLH*, *POLI*, *POLM* and *POLN* were overexpressed in CLL cells in comparison to healthy donor B lymphocytes. Unlike aforementioned DNA polymerases, *POLB* was downregulated in CLL, as well as *UBE2A* gene coding for ubiquitin-conjugating enzyme involved in the post-translational modification of replication enzymes and post-replicative DNA repair synthesis opposite of the DNA damage site.

With the exception of the DNA checkpoint gene, *CHEK1*, which was downregulated in CLL cells in comparison to healthy donor lymphocytes, most of the DNA damage response genes were overexpressed. These included genes encoding: BRCA1 that functions in replication checkpoint, maintenance of stalled replication forks and homologous recombination repair, single-stranded DNA binding protein *RPA1*, beta isoform of *TP53*, as well as *TIMELESS* and *SMARCAL1*, both of which are involved in the stabilization of DNA replication forks. The up-regulation of replication origin and DNA damage response genes in CLL may be a general adaptive response to chronic replication stress, activating replication origins to compensate for DNA replication fork stalling and inducing DNA repair to cope with chromosomal breakage.

Next, we investigated whether 3R gene expression signatures correlated to clinical features of CLL patients. Among available clinical parameters of interest (namely, time from diagnosis to initial therapy (TFT) and progression-free survival), we found that gene expression level of the replicative histone chaperone *ASF1A* could define TFT. Indeed, patients with low levels of *ASF1A* had a shorter treatment-free survival (median TFT was 12 months) than patients with intermediate and high *ASF1A* levels (median TFT was 30 months) (Figure 1d) Importantly, unlike other existing prognostic markers, *ASF1A* expression could determine TFT independently of already established clinical parameters (age, Binet staging, IGHV, 17p-, and so on) as revealed by the Cox multivariable analysis (Figure 1e, *P*<0.05). Clinical characteristics of ASF1A subgroups of CLL patients are further detailed in Supplementary Figure S4.

Histone chaperone ASF1A interacts with replicative helicase complex MCM2-7, which unwinds the double DNA helix to allow DNA polymerases to replicate single-stranded DNA and coordinates histone supply concomitantly to the course of the replication forks.^10^ Through buffering of the histone pool, governing histone post-translational modifications and directing their deposition on the chromatin, particularly in challenging conditions such as in presence of DNA replicative stress, ASF1A has a role in reestablishing the chromatin structure, and thereby, helps maintaining the chromosomal integrity and global genome stability.^11, 12^ Strikingly, histone managing driven by ASF1 has recently been implicated in the process of alternative telomere lengthening,^13^ which is readily detectable in CLL leukemic cells. Because relative telomere length has been proposed as a marker indicative for TFT,^14^ whereas arbitrarily defined telomere dysfunctions can predict CLL progression,^15^ whether *ASF1A* expression can ensure stable telomere length in CLL and reduce the risk of leukemic evolution remains an open question that needs to be further investigated.

3R gene products act as regulators of DNA metabolic processes that are essential for the stable preservation of genetic identity of every cell. CLL is a hematological disease characterized by genetic instability that can perturb controlled cellular division and motor development of cancer. Here, we investigated 3R profiles of this seemingly indolent disease and revealed strong deregulation of several genes having a role in DNA replication, repair and recombination. Finally, we show that in the 3R genetic background of CLL, *ASF1A*, which holds principal function in chromatin remodeling during DNA replication, is a novel and independent biomarker determining time to first treatment.

## Figures and Tables

**Figure 1:**
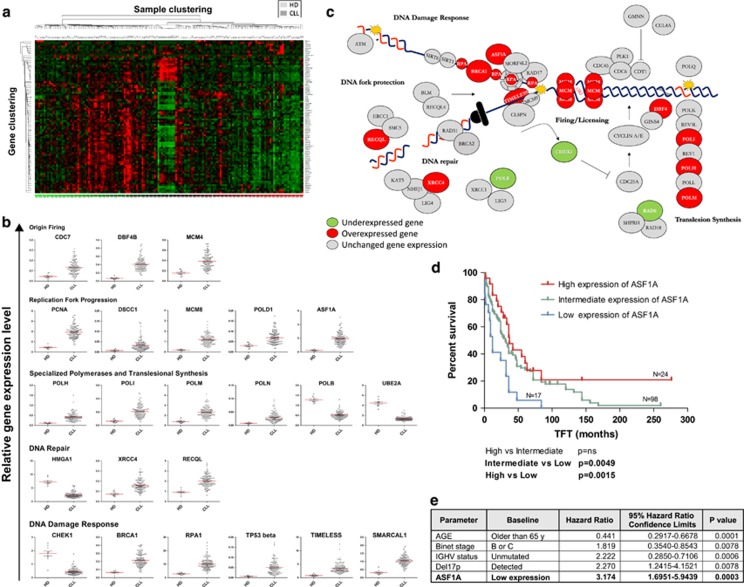
3R gene expression in CLL and ASF1A clinical implication. (**a**) Unsupervised clustering of gene expression data. (**b**) 3R genes deregulated in CLL (all *P* values <0.001). (**c**) Model of CLL replisome. (**d**) Kaplan–Meier graph of treatment-free survival according to time since diagnosis. (**e**) Multivariable analysis of time to first treatment.

**Table 1:** Patient clinical data

*Characteristic*	*Category*	n *(frequency, %)*
Sex	Female	53/136 (39)
	Male	83/136 (61)
	Unknown	5/141 (4)
Age	Younger than 65 years or 65 years old	66/137 (48)
	Older than 65 years	71/137 (52)
	Unknown	4/141 (3)
Binet stage	A	52/134 (39)
	B	54/134 (40)
	C	28/134 (21)
	Unknown	7/141 (5)
IGHV	Mutated	48/128 (37)
	Unmutated	80/128 (63)
	Unknown	13/141 (9)
Cytogenetics	Deletion 13q	41/113 (36)
	Unknown	28/141 (20)
	Trisomy 12	29/112 (26)
	Unknown	29/141 (21)
	Deletion 11q	23/137 (17)
	Unknown	4/141 (3)
	Deletion 17p	15/137 (11)
	Unknown	4/141 (3)
	Deletion 6q	8/112 (7)
	Unknown	29/141 (20)
	Complex karyotype (>3 abnormalities)	29/112 (26)
	Unknown	29/141 (20)
TP53	Mutated	7/98 (7)
	Unknown	43/141 (31)
NOTCH1	Mutated	19/96 (20)
	Unknown	45/141 (32)
SF3B1	Mutated	4/96 (4)
	Unknown	45/141 (32)

Abbreviation: IGHV, immunoglobulin heavy variable.

## Supplementary Material

Supplementary Informations
