# Supplementary material for: 3R gene expression in chronic lymphocytic leukemia reveals insight into disease evolution
Source: Blood Cancer J. 2016 Jun 3;6(6):e429–. doi: 10.1038/bcj.2016.39 (PMC5141354; doi:10.1038/bcj.2016.39)
Supplement: Supplementary Informations [file bcj201639x1.docx]

**Supplemental Materials and Methods**

***Sample preparation***

Frozen peripheral blood mononuclear cells (PBMCs) from CLL patients were provided by the Cytothèque hémopathies malignes (HIMIP). According to the French law, HIMIP collection has been declared to the Ministry of Higher Education and Research (DC 2008-307 collection 1) and obtained a transfer agreement (AC 2008-129) after approbation by technical committees (Comité de Protection des Personnes Sud-Ouest et Outre-mer II and Assistance Publique-Hôpitaux de Paris (AP-HP) ethical committee). Clinical and biological annotations have been declared to CNIL (Comité National Informatique et Libertés). Peripheral whole blood samples from healthy donors were obtained through Etablissement français du sang (EFS) (Toulouse, France).

Upon reception, CLL PBMCs were thawed out and incubated overnight at 37% in 5% CO_2_ atmosphere in RPMI media supplemented with 10% fetal bovine serum (FBS) at the concentration of 10*10^6^ cells/ml. Purification of CLL B lymphocytes from PBMC samples was performed by negative immunomagnetic selection using an EasySep Direct Human B Cell Enrichment Kit without CD43 Depletion (Stemcell Technologies, Grenoble, France).

B lymphocytes from healthy individuals were isolated directly from buffy coats using EasySep Human CD19+ Kit (Stemcell Technologies, Grenoble, France) following the manufacturer’s protocol. Purified cell populations contained >85% of B lymphocytes.

Viability of the purified cell population was estimated by staining with 7-AAD (BD Biosciences, Le Pont de Claix, France) to be >90%. qRT PCR performed on several GOIs showed no significant difference (p value > 0.4) between 2 ways of sample preparation: RNA isolation from fresh or frozen B cells. For phenotyping purposes we used following antibodies: anti-human CD5 PE-Cy7 labeled (eBioscience, Paris, France), anti-human CD19 Pacific Blue labeled (Biolegend, Saint Quantin Yvelines, France), anti-human CD3 PE-A (Beckman Coulter, Villepinte, France), anti-human CD56 PE-Cy7 (Beckman Coulter, Villepinte, France) and anti-human CD36 FITC (Immunotech, Marseille, France) along with appropriate isotypes Pacific Blue mouse IgG1, κ Isotype (Biolegend, Saint Quantin Yvelines, France), PE-Cy7 mouse IgG1 Isotype (Beckman Coulter, Villepinte, France), mouse IgG1-PE (Beckman Coulter, Villepinte, France) and mouse IgG1-FITC (Beckman Coulter, Villepinte, France) as negative controls.

***RNA extraction, first strand cDNA synthesis and quantitative Real-time PCR***

RNA was extracted from obtained samples by Trizol method using TRI Reagent (Sigma-Aldrich, Saint-Quentin-Fallavier, France). The RNA quality was determined on Agilent Bioanalyzer following the Agilent RNA 6000 Nano Kit protocol. Only samples with RNA Integrity Number (RIN) above 7 were selected for further analysis. cDNA was synthesized following a SuperScript First-Strand Synthesis protocol using dNTPs (Life Technologies, Saint-Aubin, France), Random Primer Mix (NEW ENGLAND Biolabs, Évry, France), Superscript II Reverse Transcriptase (Invitrogen, Saint-Aubin, France), RNase H (Invitrogen, Saint-Aubin, France) and RNase H Reaction Buffer (NEW ENGLAND Biolabs, Évry, France). Obtained cDNA samples were diluted to a concentration of 5 ng/μL and total sample volume of 1.5 μL was used in the preamplification step followed by a qRT PCR based gene expression analysis on a BioMark HD Reader (Fluidigm).

***Gene expression and statistical analysis***

Gene expression screening was performed on a BioMark Fluidigm platform and raw data were obtained through Fluidigm Real-Time PCR Analysis software. The assay was based on a custom selection of 94 probes including 1 internal control, probes for 7 endogenous control genes (*GAPDH, LMNB1, HUSB, IPO8, 18S, GUSB, ACTB*), 3 prognostic factors (*B2M, CLLU1, LPL*), 1 marker of proliferation (*MKI67*) and 82 3R genes of interest playing an important role in DNA replication, repair and recombination. Normalization of the expression was performed using best endogenous control gene candidates selected through geNorm algorithm.

**Table S1 Genes included in the gene expression assay**

| **Function** | **Gene name** | **Gene ID** |
| --- | --- | --- |
| **Origin firing and licensing** | Cyclin A2 | CCNA2 |
|  | Cyclin E1 | CCNE1 |
|  | Cell division cycle 45 | CDC45 |
|  | Cell division cycle 6 | CDC6 |
|  | Cell division cycle 7 | CDC7 |
|  | Chromatin licensing and DNA replication factor 1 | CDT1 |
|  | Cullin 4A | CUL4A |
|  | DBF4 zinc finger B | DBF4B |
|  | GINS complex subunit 4 (Sld5 homolog) | GINS4 |
|  | Geminin, DNA replication inhibitor | GMNN |
|  | High motility group AT-hook 1 | HMGA1 |
|  | High motility group AT-hook 2 | HMGA2 |
|  | Minichromosome maintenance complex component 2 | MCM2 |
|  | Minichromosome maintenance complex component 4 | MCM4 |
|  | Minichromosome maintenance complex component 7 | MCM7 |
|  | Minichromosome maintenance complex component 10 | MCM10 |
|  | Origin recognition somplex, subunit 4 | ORC4 |
|  | Cell division cycle 25B | CDC25B |
|  | Polo-kinase 1 | PLK1 |
|  | Bloom syndrome, RecQ helicase-like | BLM |
| **DNA replication** | Polymerase (DNA directed), alpha 1, catalytic subunit | POLA1 |
|  | Polymerase (DNA directed), delta 1, catalytic subunit | POLD1 |
|  | Polymerase (DNA directed), epsilon, catalytic subunit | POLE |
|  | Polymerase (DNA directed), theta | POLQ |
|  | CHTF18, Chromosome transmission fidelity factor 18 homolog (S. cerevisiae) | CHTF18 |
|  | DNA replication and sister chromatid cohesion 1 | DSCC1 |
|  | Fanconi anemia, complementation group M | FANCM |
|  | Minichromosome maintenance complex component 8 | MCM8 |
|  | Minichromosome maintenance complex component 9 | MCM9 |
|  | Proliferating cell nuclear antigen | PCNA |
|  | RecQ helicase-like | RECQL |
|  | RecQ protein-like 4 | RECQL4 |
|  | Structural maintenance of chromosomes 5 | SMC5 |
| **Specialized DNA polymerases  and translesional synthesis** | Polymerase (DNA directed) iota | POLI |
|  | Polymerase (DNA directed), beta | POLB |
|  | Polymerase (DNA directed), gamma | POLG |
|  | Polymerase (DNA directed), eta | POLH |
|  | Polymerase (DNA directed) kappa | POLK |
|  | Polymerase (DNA directed) lambda | POLL |
|  | Polymerase (DNA directed), mu | POLM |
|  | Polymerase (DNA directed), nu | POLN |
|  | RAD18 E3 ubiquitin protein ligase | RAD18 |
|  | REV1, polymerase (DNA directed) | REV1 |
|  | REV3-like, polymerase (DNA directed), zeta, catalytic subunit | REV3L |
|  | SNF2 histone linker PHD RING helicase, E3 ubiquitin protein ligase | SHPRH |
|  | Ubiquitin-conjugating enzyme E2A | UBE2A |

| **Function** | **Gene name** | **Gene ID** |
| --- | --- | --- |
| **DNA repair** | Breast cancer 1, early onset | BRCA1 |
|  | ERCC1 | ERCC1 |
|  | Ligase III, DNA, ATP-dependent | LIG3 |
|  | Ligase IV, DNA, ATP-dependent | LIG4 |
|  | Nonhomologous end-joining factor 1 | NHEJ1 |
|  | X-ray repair complementing defective repair in Chinese hamster cells 1 | XRCC1 |
|  | X-ray repair complementing defective repair in Chinese hamster cells 4 | XRCC4 |
|  | X-ray repair complementing defective repair in Chinese hamster cells 4 (isoform 1) | XRCC4 iso 1 |
|  | RAD51 recombinase | RAD51 |
|  | Breast cancer 2, early onset | BRCA2 |
| **DNA damage response (DDR)** | Anti-silencing function 1A histone chaperone isoform a | ASF1A |
|  | Claspin | CLSPN |
|  | E1A binding protein p300 | EP300 |
|  | Fanconi anemia, complementation group D2 | FANCD2 |
|  | K(lysine) acetyltransferase 5 | [KAT5](http://www.ncbi.nlm.nih.gov/entrez/query.fcgi?db=gene&cmd=Retrieve&dopt=Graphics&list_uids=10524) |
|  | Mortality factor 4 like 1 | f4L1 |
|  | Mortality factor 4 like 2 | MORF4L2 |
|  | Protein kinase, DNA-activated, catalytic polypeptide | PRKDC |
|  | RAD17 homolog (S.pombe) | RAD17 |
|  | RAD9 homolog A (S.pombe) | RAD9A |
|  | Replication protein A1, 70 kDa | RPA1 |
|  | SIN3 transcription regulator family member B | SIN3B |
|  | Sirtuin 1 | SIRT1 |
|  | Sirtuin 6 | SIRT6 |
|  | SWI/SNF related, matrix associated, actin dependent regulator of chromatin, subfamily a-like 1 | SMARCAL1 |
|  | Timeless circadian clock | TIMELESS |
|  | Topoisomerase (DNA) II binding protein 1 | TOPBP1 |
|  | Tumor protein p53 | TP53 |
|  | Tumor protein p53 (isoform beta) | TP53 beta |
|  | ATM serine/threonine kinase | ATM |
|  | ATR serine/threonine kinase | ATR |
|  | Checkpoint kinase 1 | CHEK1 |
|  | Checkpoint kinase 2 | CHEK2 |
|  | Helicase, POLQ-like | HELQ |
|  | Mbt domain containing 1 | MBTD1 |
|  | PHD finger protein 12 | PHF12 |
| **CLL diagnostic  markers** | Beta-2-microglobulin | B2M |
|  | Chronic lymphocytic leukemia up-regulated 1 | CLLU1 |
|  | Lipoprotein lipase | LPL |
| **Proliferation  marker** | Marker of proliferation Ki-67 | MKI67 |
| **Endogenous  control genes** | RNA, 18S ribosomal 5 | 18S |
|  | Actin, beta | ACTB |
|  | Glucuronidase beta | GUSB |
|  | Glyceraldehyde-3-phosphate dehydrogenase | GAPDH |
|  | Hydroxymethylbilane synthase | HMBS |
|  | Importin 8 | IPO8 |
|  | Lamin B1 | LMNB1 |

**COLOR LEGEND**

| **Genes** affected by ... | **Functional partners** of  genes affected by … |
| --- | --- |
| 17p deletion | 17p deletion |
| 11q deletion | 11q deletion |
| Trisomy 12 | Trisomy 12 |
| 13q deletion | 13q deletion |
| Low penetrance gene | Functional partner of a  low penetrance gene |

**Table S2 Summarized 3R genes deregulated in CLL**

|  | **Origin firing** | **Replication fork progression** | **Specialized polymerases  and translesional synthesis** | **DNA repair** | **DNA damage response** |
| --- | --- | --- | --- | --- | --- |
| **Upregulated** | CDC7 | ASF1A | POLH | RECQL | TP53beta |
|  | DBF4B | DSCC1 | POLI | XRCC4 | RPA1 |
|  | MCM4 | MCM8 | POLM |  | SMARCAL1 |
|  |  | PCNA | POLN |  | TIMELESS |
|  |  | POLD1 |  |  |  |
| **Downregulated** |  |  | POLB | HMGA1 | CHEK1 |
|  |  |  | UBE2A |  |  |

**Figure S1 CLL cohort validation (Kaplan-Meier estimates)** (A) IGHV mutational status determines time to first treatment (TFT). (B) Presence of complex karyotype (CK>3 chromosomal abnormalities) predicts progression free survival. (C) CLLU1 gene expression predicts progression free survival (PFS).

A

**TFT**

according to **IGHV mutational status**

Mutated IGHV

Unmutated IGHV

B

**PFS**

according to **complex karyotype**

(>3 chromosomal abnormalities)

Karyotype < 3 abnormalities

Karyotype > 3 abnormalities

**Figure S2 Differential 3R gene expression in CLL subgroups** (A) According to the presence of a single karyotypic abnormality. B) According to the IGHV mutational status. C) According to the presence of a TP53 mutation. D) According to the presence of a NOTCH1 mutation.

C

**PFS**

according to **CLLU1 expression**

CLLU1 not overexpressed

CLLU1 overexpressed

**A) Karyotipic abnormality**

**Trisomy 12**

**Deletion 17p**

**B) IGHV mutational status**

**C) TP53 mutational status**

**D) NOTCH1 mutation**

**Figure S3 Characterization of individual CLL clusters.** A) Gene expression based clustering of CLL samples. B) Clinical characteristics of single CLL clusters.

A
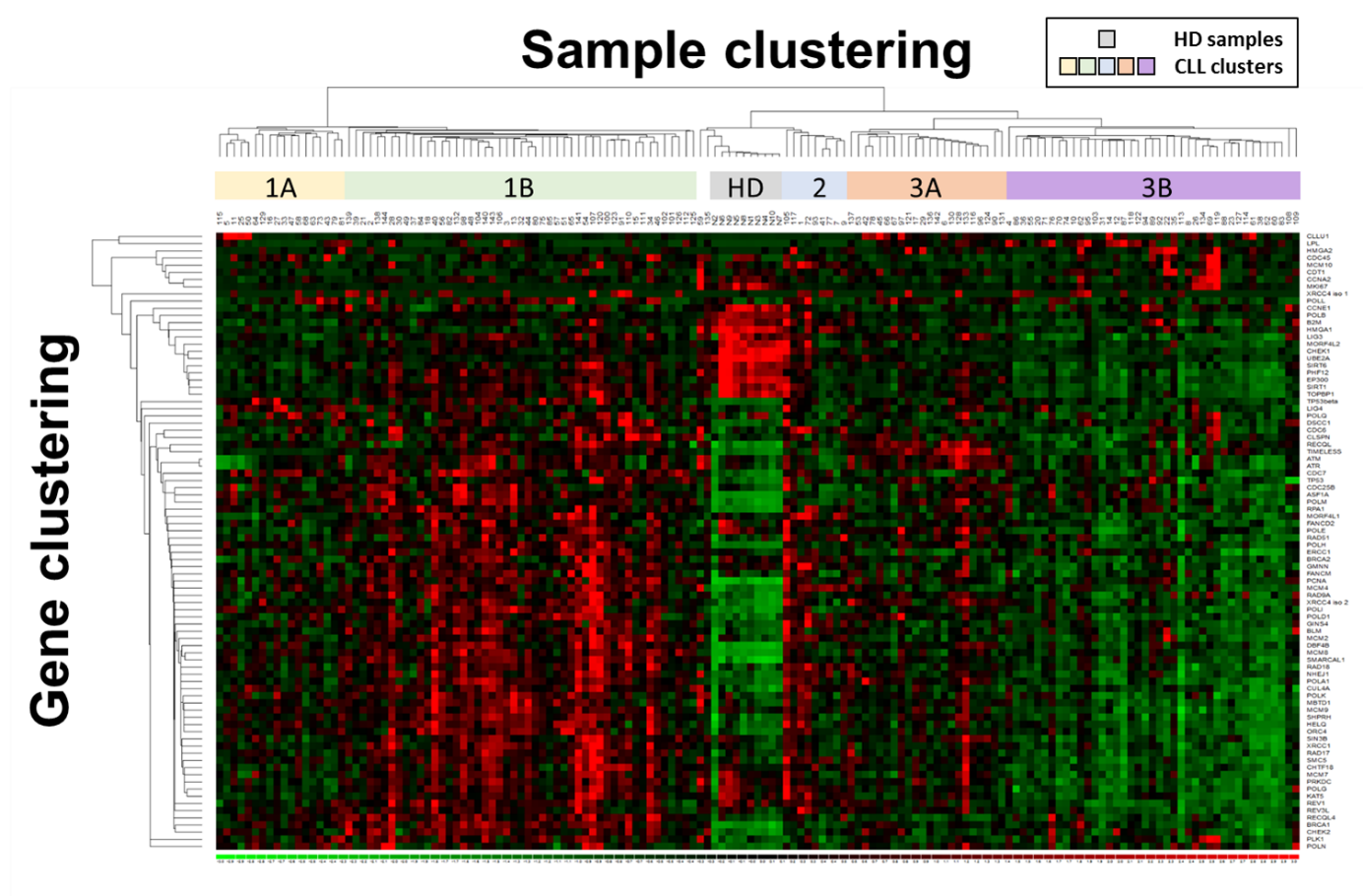


B

|  | **CLL cluster** | | | | |
| --- | --- | --- | --- | --- | --- |
|  | **1A** | **1B** | **2** | **3A** | **3B** |
| **Number of  patients/cluster** | 18 | 49 | 9 | 22 | 41 |
| **Gender (M, F; %)** | 65, 35 | 57, 43 | 86, 14 | 64, 36 | 59, 41 |
| **Age (mean; years)** | 69 | 65 | 67 | 66 | 64 |
| **Binet (A, B, C; %)** | 35,47,18 | 47,36,17 | 29,29,43 | 18,68,14 | 44,31,26 |
| **IGHV (M, UM; %)** | 67, 33 | 41, 59 | 71, 29 | 90, 10 | 76, 24 |
| **SF3B1** | 0/14 | 0/33 | 0/5 | 3/15 | 1/29 |
| **NOTCH1** | 2/14 | 1/33 | 2/5 | 7/15 | 7/29 |
| **TP53** | 0/14 | 2/33 | 1/6 | 1/15 | 2/29 |
| **TRI12 (%)** | 0 | 5 | 25 | 79 | 31 |
| **DEL11Q (%)** | 33 | 17 | 0 | 5 | 17 |
| **DEL13Q (%)** | 50 | 45 | 0 | 11 | 40 |
| **DEL17P (%)** | 6 | 4 | 29 | 14 | 15 |

**Figure S4 Clinical features of patient subgroups according to ASF1A gene expression.**

|  | **ASF1A low** | **ASF1A intermediate & high** | **p value** |
| --- | --- | --- | --- |
| 17p deletion | 25% (5/20) | 9% (11/117) |  |
| TFT (mean; months)  for 17p- patients | 41 | 17 | 0,072 |
| TP53 mutation | 14% (2/14) | 6% (5/86) |  |
| TFT (mean; months)  for TP53 mutated patients | 24 | 22 | 0,92 |
| 11q deletion | 10% (2/20) | 18% (21/117) |  |
| TFT (mean; months)  for 11q- patients | 54 | 31 | 0,39 |
| Binet stage A | 32% (6/19) | 40% (46/115) |  |
| TFT( mean; months) for Binet A patients | 34 | 52 | 0,37 |
| Binet stage B | 37% (7/19) | 40% (49/115) |  |
| TFT( mean; months) for Binet B patients | 7 | 44 | 0,072 |
| Binet stage C | 31% (6/19) | 19% (22/115) |  |
| TFT( mean; months) for Binet C patients | 28 | 35 | 0,72 |
